# Primary Care Support Tools for Digestive Health Care: A Mixed Method Study

**DOI:** 10.1155/2024/6805365

**Published:** 2024-07-24

**Authors:** Mubashir Arain, Leanne Reeb, Rebecca C. Miyagishima, Julia Carter, Kerri L. Novak

**Affiliations:** ^1^ Health Systems Knowledge & Evaluation Alberta Health Services, Calgary, Canada; ^2^ Digestive Health Strategic Clinical Network Alberta Health Services, Edmonton, Canada; ^3^ Division of Gastroenterology and Hepatology University of Calgary, Calgary, Canada

## Abstract

**Background:**

To address the increasing demands for gastroenterology specialty care and increasing wait times, centralized access and triage (CAT) systems, telephone support, and clinical care pathways were implemented to streamline referrals and support management of low-risk gastrointestinal (GI) conditions in the primary care medical home. This study aimed to understand primary care providers (PCPs) and GI specialists' perceptions of these supports, factors that affect support implementation and identify barriers and facilitators for implementing supports from both PCP and GI specialists' perspectives.

**Methods:**

We conducted a mixed method study including surveys and interviews with PCPs and GI specialists. Online surveys and semistructured qualitative interviews were conducted from July 2022 to September 2022. All interviews were transcribed and coded to perform a thematic analysis. Survey data were analyzed in SPSS version 25. Descriptive statistics were employed to summarize and describe the data collected. Inferential statistics were used to identify associations and relationships within the dataset. *T*-test and chi-square tests were applied at 95% confidence level, with a *p* value <0.05 (two-sided) considered statistically significant.

**Results:**

A total of 36 PCPs responded to the survey. Most respondents were working full-time (73.5%, *n* = 25) and were female (73.5%, *n* = 25). Overall, 42% used the pathways regularly, 48% (*n* = 16) used them occasionally, and very few (9.1%, *n* = 3) said they were aware but had not used pathways. Overall, PCPs were satisfied with CAT processes and the use of primary care pathways, recognizing the importance of fair and equitable access to specialty care. Specific processes in CAT for vulnerable populations and patients using walk-in clinics were recognized as a limitation, given the lack of ease in completing the required testing and follow-up needed when utilizing the care pathway. Of the 112 GI specialists who received the survey, 28 (25%) completed it, with males (50.0%, *n* = 14) and females (39.2%, *n* = 11), remainder no response. Most participate in CAT (73.9%, *n* = 17) and were remunerated by an alternative relationship plan (ARP) (53.6%, *n* = 15). Overall, GIs were satisfied with central triaging and primary care pathways, reducing unnecessary time and resource expenditure for referrals. There were statistically significant differences in perceptions among fee for service and alternative relationship plan GI specialists regarding the effectiveness of CAT in improving access and use of health system resources.

**Conclusion:**

Overall, PCPs and GI specialists believe utilizing CAT and primary care pathways improves referral quality, reduces resource expenditure, and provides fair and equitable access to GI specialty services. Improvement in CAT processes with improved pathway awareness may reduce unnecessary referrals.

## 1. Introduction

Digestive disorders and related conditions impact the health and quality of life of many Canadians. A recent survey of global prevalence and burden of functional gastrointestinal (GI) conditions showed 41.3% (95% CI: 39.1–43.4) of Canadians surveyed (*N* = 2029) reported having a functional GI disorder, slightly higher than the worldwide prevalence of 40.3% (95% CI: 39.3–40.7) [1]. Colorectal cancer is currently the 3rd most diagnosed type of cancer among Canadians, affecting 1 in 14 men and 1 in 18 women [2, 3]. Furthermore, the burden of digestive disorders and diseases on both Canadians and provincial health systems is expected to increase as a large proportion of the population ages [4].

The best-available Canada-wide data on wait times for gastroenterology and related care come from the now dated 2012 SAGE Survey of Access from the Canadian Association of Gastroenterology (CAG) estimating a median wait time for consultation of 92 days (95% CI: 85–100). The median wait time for procedures was 55 days (95% CI: 50–61), resulting in a total median wait time of 155 days (95% CI: 142–175). Average and median wait times vary significantly between provinces due to factors specific to the provincial health systems (macro) as well as individual patient and cultural differences (micro) [4]. Similarly, provision of gastroenterology care is not uniform throughout the country. Telford et al. [5] report the most common method of managing referrals to gastroenterology clinics is a “first-in/first-out, often with an ad hoc prioritization for urgent cases,” while some provinces have implemented other methods of triaging patients using various “prioritization tools.” Moreover, Switzer et al. [6] note that general surgeons perform nearly half of all colonoscopies in Canada, particularly in Prince Edward Island and Manitoba. While gastroenterologists are reported to perform a significant proportion of endoscopies in Alberta, other providers, such as general internists, primary care physicians (PCPs), and surgeons perform endoscopic procedures as part of their practice [6] Across Canada, the COVID-19 pandemic reduced capacity in both primary and specialty care practices, increased referral backlog, and adversely impacted health outcomes of patients facing longer wait times for health services. For example, one Ontario-based study found a 38% decrease in endoscopic procedures for patients with inflammatory bowel disease (IBD) between 2019 and 2020 [7]. The COVID-19 pandemic also limited training opportunities for gastroenterology trainees when shortage of gastroenterologists in Canada is a recognized barrier for patients with digestive health concerns. Khan et al. [8] reported a statistically significant decrease in procedures performed between the pre-COVID period and during the pandemic, thus contributing to longer training requirements and reduced clinical competency.

The province of Alberta, situated in the western part of Canada, boasts a diverse and dynamic healthcare landscape. As one of the fastest-growing provinces in the country, Alberta has faced unique challenges in maintaining and improving healthcare access for its residents. In Alberta, patients with digestive health symptoms and conditions often face difficulty accessing specialty care within an appropriate timeframe. Long wait times exist for both GI specialist consultations as well as endoscopy services due to a high volume of referrals and limited endoscopy suite capacity [9], according to [10]. For patients categorized as “routine,” wait times are a minimum of 12–24 months, which may result in lowered quality of life and adverse health outcomes.

Alberta has focused the restructuring of primary health to build a patient-entered medical (PCM) home, aimed to provide significant benefit to patients with chronic conditions through collaborative multidisciplinary care guided by their family physician. This collaborative team-based, integrative care occurs within a primary care network (PCN) [11]. Codeveloped clinical care pathways (specialty and primary care) implemented for a number of conditions support care within primary care and optimize referral appropriateness to specialty care [12]. In addition, support is available with specialist physicians by telephone through the specialist link program [13] and connect MD [14] and through electronic advice.

Clinical care pathways are used in Canada and throughout the world, as a tool to implement best-evidence guidelines into practice [15]. Care pathways function as “recommendations for optimal management plans” utilizing concrete “sequences” of evaluation and timing for specific testing and treatment [15]. Care pathways exist in various sectors of clinical care and for myriad conditions and can be an effective means of translating evidence-based practice into clinical care [16]. Myriad outcome benefits of pathways include the reduction of adverse events, mortality rates, decreased wait times for healthcare services, and specialty care, among others [15]. One Canadian study evaluating the impact of an acute care surgery pathway for appendicitis reported decreases in wait time from emergency department (ED) triage to surgery, sustained at 12-month follow-up [17]. An Alberta-based study evaluating a perioperative glycemic management quality improvement pathway found use of the pathway increased screening rates as well as A1C testing for patients [18]. There are nine clinical pathways for GI conditions in use in Alberta including chronic abdominal pain, chronic constipation, chronic diarrhea, dyspepsia, gastroesophageal reflux disease (GERD), H. pylori, hepatitis C, irritable bowel syndrome (IBS), and nonalcoholic fatty liver disease (NAFLD) [12].

Alongside the GI pathways, gastroenterology care in the urban centers in Alberta is provided through a centralized access and triage (CAT) system. The foundation of the CAT system occurred in Calgary, Alberta, in 2005, within a community of academic gastroenterologists [20]. This innovation starkly contrasted a previous system in which practitioners managed their own referrals and rosters on an individual basis. The proposed benefits of the CAT system include increased access, reduction in wait times as well as enhanced system knowledge which can better respond to areas of demand, gaps in provision of services or access, and other challenges. Use of the CAT system spread to Edmonton's University of Alberta Hospital in 2009 and throughout the province in the following years (Novak et al. 2013).

This paper will demonstrate the following:

- PCPs and GI specialists' perceptions of primary care supports
- Barriers and facilitators to implement primary care supports, from both PCP's and GI specialist's perspectives

## 2. Methods

A mixed method approach was used to explore PCPs and GI specialists' experiences and satisfaction with the primary care support tools. Data collection took place between March and September 2022. This study was approved by the University of Calgary Conjoint Health Research Ethics Board (REB19-2106). All participants were provided with information on the project and how the data would be used.

### 2.1. Surveys

The survey was developed by the authors in consultation with key stakeholders including PCPs and GI specialists. It was pilot tested on a small sample before being formally rolled out, with no changes required after pilot testing. The survey was developed and implemented using Select Survey. A different version was used for PCPs and GI specialists. PCP participants were recruited through two main avenues: firstly, survey invitation letters were distributed during a primary care conference and secondly, survey invitation letters were attached to referral closures letters from CAT centers in Calgary and Edmonton. GI specialists were recruited through a dissemination of survey invitation letters through the Digestive Health Strategic Clinical Network (DHSCN) and through the project lead. The survey ran from March to June 2022 for both GI specialists and PCPs. A draw was also included at the end of the survey for a $500 gift card. The sampling frame included 112 Albertan GI specialists who received the online survey. The number of PCPs is unknown due to the methods of PCP recruitment. To increase the response rate, we used QR codes on the invitation to scan directly from the online survey. The invitation letter included a brief description on the project and link/QR code to the survey. Participation was voluntary and all information provided was anonymous and confidential. Only participants who expressed their interest to participate in a qualitative interview and provided their contact information were approached.

Survey data were analyzed in IBM SPSS version 25. Descriptive and inferential statistics were used to provide data summaries and associations. *T*-test and chi-square tests were applied at 95% confidence level; *p* value <0.05 was considered as significant. Responses to open-ended survey questions are provided under each section of PCP and GI specialist survey findings.

### 2.2. Interviews

Survey responders who expressed interest in participating in a telephone interview were approached by the evaluation team. Seven GI specialists and 20 PCPs agreed to be contacted for the interview with 19 PCP and 7 GI specialist interviews completed. Interviews were conducted from July 2022 to September 2022, each lasting 30 minutes. Participants were offered a $50 gift card as a token of appreciation. Semistructured interview guides were used; slightly different versions for PCPs and GI specialists. The interview guide was developed along with the survey. Due to restrictions from the Ethics Board, the survey data were not linked to the qualitative interviews as the identifiers were deleted from the survey. Interview guide questions for GI specialists included participation and perceptions about CAT, awareness of primary care pathways, and challenges with wait times for GI referrals and endoscopies. The interview guide for PCPs included awareness and utilization of primary care pathways, perceptions about the benefits of pathways and central triage services, and the challenges with wait times for GI specialist referrals and endoscopies.

Most interviews were conducted over MS Teams, a few were over the telephone. With permission from the participants, we also recorded all interviews to ensure accuracy during write-up and analysis. After removing identifiable information, each set of verbatim interview transcripts was cleaned for time stamps, repeated words, or inaccurate words inserted by transcription recording software. An evaluation team member compared the audio file with the written transcript to confirm accuracy of content. Two authors (MAA and RM) followed qualitative analysis processes, coded transcripts, and then compared themes that emerged. During this process, overarching main themes were named and described. Between team members, the main themes were agreed upon. Once all members had completed theming, one team member merged all working copies into a master file. All files were stored on the secure server.

Due to the voluntary nature of participation in both the survey and interviews, PCP and GI samples consisted of self-selected individuals.

## 3. Results

### 3.1. Survey Findings

#### 3.1.1. Primary Care Provider Survey

A total of 36 PCPs responded to the survey. The majority of respondents were from Calgary (61.1%, *n* = 22) and Edmonton (25.0%, *n* = 9) (Table 1). Most reported (86%, *n* = 31) being a member of a PCN, most reported being in practice for 6–10 years (38.2%, *n* = 13), and most worked full-time (73.5%, *n* = 25). The majority of PCPs surveyed were female (73.5%, *n* = 25).

**Figure 1:**
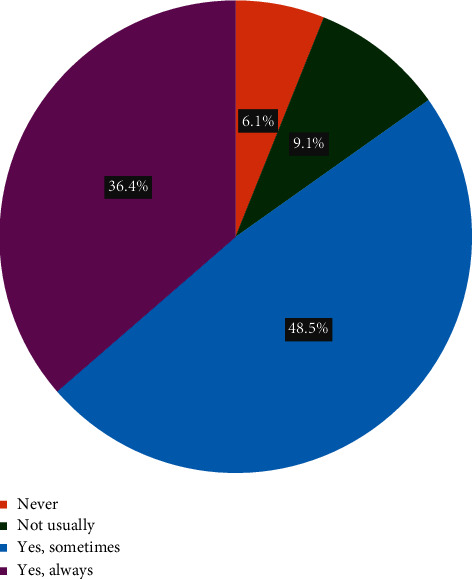
Usefulness of primary care pathways in practice (*N* = 33).

Around 42% (*n* = 14) of participants said they were using the pathways regularly. Roughly 48% (*n* = 16) used them occasionally while very few (9.1%, *n* = 3) were aware of the pathways but had not used them. Around 36.4% (*n* = 12) of respondents always found primary care pathways useful, while 48.5% (*n* = 16) only sometimes found them useful (Figure 1). Few (6.1%, *n* = 2) reported never finding the pathways useful, while 9.1% (*n* = 3) rarely found the pathways useful.

**Figure 2:**
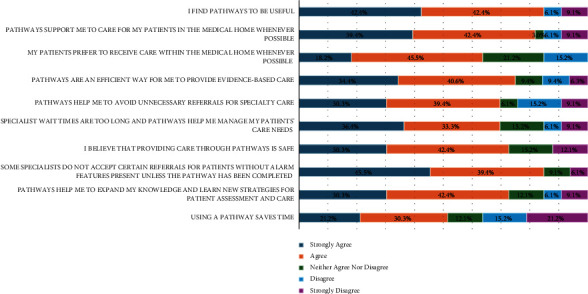
Reasons for choosing to use a primary care pathway (*N* = 33^*∗*^). ^*∗*^The statement “pathways are an efficient way for me to provide evidence-based care” had 32 (*n* = 32) respondents.

Most respondents strongly agreed (42.4%) or agreed (42.4%) pathways were useful (Figure 2) and felt they supported PCPs to care for patients in the PCM (39.4% strong agreement and 42.4% agreement). The largest proportion of respondents strongly agreed (45.5%) or agreed (39.4%) pathways were used because GI specialists would not accept their referral. The largest proportion of disagreement from respondents was for the statement “Using a pathway saves time” with 15.2% disagreed and 21.2% strongly disagreed.

**Figure 3:**
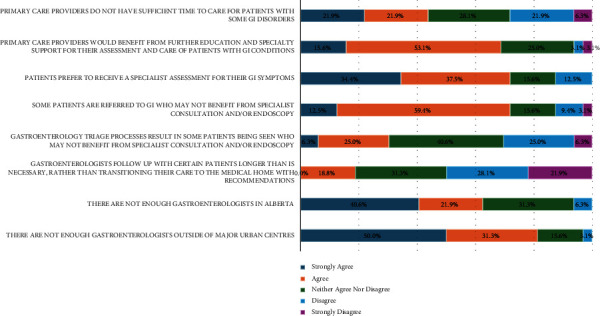
Factors contributing to wait times for access to GI specialty care (*N* = 32).

Respondents strongly agreed patients preferred to see a GI specialist for assessment of their GI symptoms (34.4%), and lack of GI specialists outside of major urban centers (50.0%) contributed to wait times for access (Figure 3). The largest proportion of disagreement was for the statement, “Gastroenterologists follow up with certain patients longer than necessary, rather than transitioning their care to the medical home with recommendations” (28.1% disagreed and 21.9% strongly disagreed). In addition, most respondents were neutral as to whether gastroenterology triage results in patients being seen who may not benefit from either GI specialist consultation and/or endoscopy (40.6% neither agreed nor disagreed).

#### 3.1.2. GI Specialist Survey

There were 28 out of 112 GI specialists who completed the survey (response rate of 25%). Most (60.7%, *n* = 17) practiced in Calgary with nearly equal gender, males (50.0%, *n* = 14) and females (39.2%, *n* = 11) (Table 2). Most had been in practice for 6–10 years (28.6%, *n* = 8), and the majority had a full-time practice (96.4%, *n* = 27) and were participating in a CAT (73.9%, *n* = 17). The most cited remuneration model was the ARP∗ (53.6%, *n* = 15), followed by fee-for-service (42.9%, *n* = 12) (Table 2).

**Figure 4:**
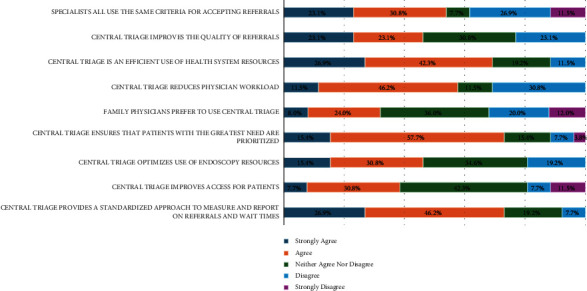
Benefits of CAT (*N* = 26^*∗*^). ^*∗*^The statement “family physicians prefer to use central triage” had a total of 25 (*N* = 25) respondents.

The majority of respondents believed CAT was an efficient use of healthcare resources (26.9% strongly agreed and 42.3% agreed) and CAT ensures those with the greatest need are given high priority (15.4% strongly agreed and 57.7% agreed) (Figure 4). In addition, respondents reported CAT reduced physician workload (11.5% strong agreement and 46.2% agreement) and provided standardized means of measuring and reporting on referrals and wait times (26.9% strong agreement and 46.2% agreement). However, respondents were not convinced that CAT improved the quality of referrals (30.8% neither agreed nor disagreed) or access for patients (42.3% neither agreed nor disagreed).

**Figure 5:**
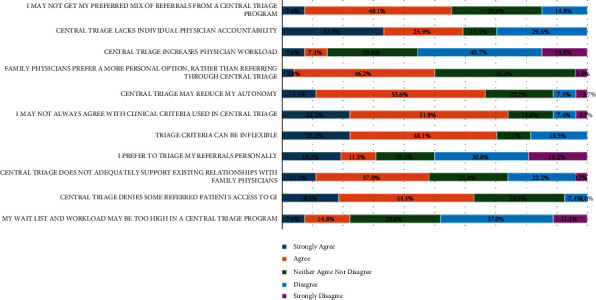
Disadvantages of CAT (*N* = 27^*∗*^). ^*∗*^The statements “I prefer to triage my referrals personally” and “family physicians prefer a more personal option, rather than referring through central triage” each had a total of 26 (*N* = 26) respondents.

A large proportion of respondents believed participation in CAT reduced their autonomy (11.1% strongly agreed and 55.6% agreed) (Figure 5). Two contributing factors to this sense of loss of autonomy were lack of agreement with clinical criteria used in CAT (22.2% strongly agreed and 51.9% agreed) and inflexibility of CAT criteria (22.2% strongly agreed and 48.1% agreed). However, most respondents disagreed that CAT programs increased their workload (40.7% disagreed and 14.8% strongly disagreed) or that CAT rendered their workload too high (37.0% disagreed and 11.1% strongly disagreed).

*(1) Comparison between GI Specialist on FFS and ARP Models*. Significant variations were seen for FFS and ARP GI specialists regarding their views about the benefits of CAT services and primary care pathways. FFS model specialists were significantly less likely to be involved in CAT (Table 3), while ARP specialists were significantly more likely to strongly agree/agree to the statements that “CAT is an efficient use of health system resources” and “CAT improves access for patients” (Table 3).

Table 4 compares the perceptions of ARP and FFS specialists regarding views about pathway development and utilization. ARP specialists were significantly more likely to agree with the statement “pathways were codeveloped between primary and specialty care providers” (Table 4), and they had significantly more positive responses towards pathways such as “all specialties use a common format for pathways” and “pathways across all specialties are available in a single location” (Table 4).

### 3.2. Qualitative Interviews

#### 3.2.1. Primary Care Provider Interviews

We conducted 19 interviews with PCPs. Three PCPs had <5 years of PCP experience, while all others had 5 or more years of experience. There were 13 PCPs working full time. The following are key themes that emerged from the PCPs' interviews.

*(1) Awareness of Primary Care Pathways*. All interviewed PCPs were aware of primary care pathways and most regularly used them. PCPs mentioned the use of pathways not only helped to provide the best care to patients in the medical home, but also to ensure appropriate referrals were sent to the GI specialists.

*I think pathways are good because they follow the evidence-based practice. They give some things that you should consider, so maybe it will slow down the number of referrals needed for things that can be managed in primary care. [PCP, Edmonton]*

However, some PCPs were aware of the pathways but not utilizing them fully in their practice.

*I think that they [Pathways] are potentially very helpful, but I probably haven't implemented them fully yet in my practice. [PCP, Edmonton]*

It was mentioned that higher awareness of the GI pathways would be helpful.

*I think just raising more awareness of the breadth of the pathway. So I think you know I've looked at the pathways before, I'm familiar with the Specialist Link, but even for me, I didn't know that there were so many different pathways as existed for GI. [PCP, Calgary]*

One participant appreciated the educational sessions conducted to increase awareness of primary care pathways.

*Probably people who haven't done their due diligence and gone through the pathways when they get that back, probably get very frustrated. But again, I think that's like a medical education thing and hopefully, like the series that Dr. XX and her team did, if you know if it reaches more people, more people will be aware and then we will be sending less referrals and that will help. [PCP, Calgary]*

*(2) Perceived Impact of Primary Care Tools on Reducing Inappropriate Referrals*. Participants perceived a significant positive impact with the implementation of primary care supports such as pathways and telephone advice. During the interviews, one Calgary PCP provided an example where the patient was managed in a medical home.

*I use the pathway for chronic diarrhea. He was a patient that came to me and I had worked him up a little bit, but I thought that he was a, you know, in the age group where with his problem he might need a colonoscopy. His referral was rejected at the beginning. I did follow the pathway and determined he had the celiac disease after that. So, it prevented him from needing a colonoscopy. [PCP, Calgary]*

Similarly, a PCP from Edmonton also perceived a positive impact of ConnectMD on reducing inappropriate referrals.

*I think having our phone access to connect MD and I think it's maybe Specialist Link in Calgary, which is like the phone consult, I think that has been great. Probably helps to alleviate because sometimes referrals are just…. You're not really sure what the next step is or what you should do, or if it needs a referral or needs to be seen. So, a lot of those kinds of more ambiguous referrals can be taken care of effectively through the connect MD. [PCP, Edmonton]*

*(3) Perceptions about the Benefits of Primary Care Pathways*. Participants spoke of the benefits of primary care pathways.

*I find them [Pathways] very useful. I could just even use dyspepsia as an example. You know there sometimes with dyspepsia, we might not, initially because there's so much you're thinking about all at once, and you've only got 10 minutes for an appointment. So, your brain is trying to think of all the different things that we should be thinking of all at once. And sometimes we tend to miss something simple. [PCP, Edmonton]*

*I think Specialist LINK integrating with them [Pathways] is a really good idea. I think that, you know, needs to be there so that you can kind of say oh, maybe they don't need to phone Specialist LINK. I'll just look at the pathways. [PCP, Calgary]*

There was an example provided by IBS pathways which the PCP found very useful in patient management.

*Irritable bowel syndrome has also been a good tool. I find there's often a lot of anxiety for people with that diagnosis and fear that it's something else so going through the pathway can be very helpful to reassure the patient that we're managing their symptoms properly. [PCP, Calgary]*

*(4) Challenges with Primary Care Pathways*. One of the PCPs from Calgary explained the number of diagnostic tests that are required to complete for pathways sometimes goes against the Choosing Wisely Canada guidelines.

*If there's one piece of information missing, the whole referral is rejected, and then you have to get that and send it back the other challenge I have with that is that a lot of the, I'm quite interested in choosing Wisely Canada and some of their guidelines and a lot of the things that are recommended were insisted on by triage are actually contrary to what Choosing Wisely recommends, so we're maybe doing unnecessary tests and then we're responsible for those for the 2 1/2 years while the patients waiting to see the specialist [laughs] is very, [laughs] very, very challenging. [PCP, Calgary]*

Another PCP from Edmonton mentioned a very similar concern about ordering too many diagnostic tests to complete the pathways.

*I think they [pathways] are good. They're the fatty liver ones, pretty comprehensive, and kind of the workup that's needed for every patient. And you know, sometimes I feel like oh, this is way more than I would have ordered on most patients with just normal ALT for example. So that sometimes I wondered, am I like costing the system a lot more by doing every test on these patients? [PCP, Edmonton]*

Participants were concerned about completing the pathways for walk-in patients which they found very challenging.

*I found walk-in patients even more difficult and I cannot imagine having to do pathways. If I was doing a walk-in clinic. I mean, walk-in clinics are so rapid-paced and just so like there's no time to be doing extra pointing and clicking. [PCP, Edmonton]*

*(5) CAT Streamlining the Referral Process*. The implementation of CAT streamlines the referral process.

*The process has been more streamlined with it [CAT] and then also with the presence of Specialist Link. It is supposed to centralize things and make them more equal and standardized across the city, so it feels like it's fair. So you don't feel like some patients are getting into seeing certain specialists and others are not… it's all shared. [PCP, Calgary]*

*I really like central access systems. I feel like that's a better way of triaging and getting to the right person faster rather than not being aware of all the different specialists and having to pick and choose one from a group of gastroenterologists. I like central access. [PCP, Calgary]*

*(6) Challenges with CAT*. PCPs also expressed frustration with CAT and believed that there could be a way for PCPs to get faster access when a PCP strongly feels a patient needs to be seen by a GI specialist quickly.

*If we are sending a really urgent referral, there are red flags. It's a legitimate reason for referral and you know our office of calling your central triage and trying to sort this out then to be told, Oh well, this is going to take several months, and then in several months being told, well, it'll still take several months, things like that shouldn't really be happening. [PCP, Calgary]*

One PCP mentioned that she started using CAT but after seeing a number of inappropriate refusals (i.e., a discrepancy between the referring PCP and CAT resulting in refusal of the referral), she stopped using the central triage in Calgary. Particularly for referrals requiring urgent attention, PCPs requested better referral processes to expedite urgent cases.

*I don't know if there's a better way to sort of have a, Is this quote urgent, and why on the form? Or is this routine or is this semi-urgent and why so that it puts the onus on the referring physician to explain why they feel it's urgent and maybe all those urgent check marks? Urgent referrals could be looked at first. [PCP, Calgary]*

Another PCP emphasized the need for better standardized processes for the referral.

*I think that a standardized referral form would be much better than a pathway so that the pathway means that have you done these tests and if you have written information on your referral letter which corresponds to that pathway, the referral gets accepted. [PCP, Calgary]*

*(7) Education and Training*. Participants agreed that additional education and training would make PCPs more comfortable and confident in caring for GI-related conditions and processes.

*Some education for family doctors and teams will be helpful to see what's happening within the gastroenterology world because some of us did our training quite a long time ago, so having those, and I mean the pathways are part of that, but having some more education to enable us to do a little bit more within primary care would be good. [PCP, Calgary]*

Also, more communication in the form of feedback from GI specialists regarding the referral quality would be helpful for PCPs.

*We just don't have enough communication between the two groups where they say to us, look, we're doing our best, we are so overwhelmed, we have 200 referrals a day; there's no way we can look at them all and you know it makes us then think am I over referring or am I providing enough information or am I providing too much information. [PCP, Calgary]*

A periodic clinical audit of referrals could be useful to use as an educational tool for improving the quality of referrals.

*I think there's a huge area for some education back to the referrers and the referring people could provide us with a little bit of [feedback], you know and hopefully, I think that could go a long way in improving the quality of referrals. [PCP, Calgary]*

#### 3.2.2. GI Specialist Interviews

A total of seven GI specialists participated in the interviews. Three had <5 years of experience in GI, while the others had 6 or more years of experience. Four GI specialists were remunerated through FFS and 3 ARP/AMHSP. There were 5 GIs who regularly participated in CAT services, while two had never participated.

There was significant response variation between the GI specialists working on FFS versus ARP/AMHSP models regarding the benefits of central triage, primary care pathways, and other tools. The following are key themes that emerged from the seven interviews.

*(1) Pathways and Appropriate GI Specialist Referrals*. Participants agreed that there are many benefits to the pathways. According to one GI specialist, pathways provide a guideline for PCPs to consider when thinking about making a GI referral.

*There are many patients that I think could be realistically managed in their medical home with their family practitioner. The pathway would help identify those patients by giving the general practitioners a path to follow, to order the appropriate tests, excluding other diagnoses, and for assistance with management. [GI specialist, Fee for Service]*

*I've been working in Calgary for about three years. So, I think the uptake [of using the pathways] has increased. Maybe 75% of my patients have completed pathways when I see them. [GI specialist, Fee for Service]*

All GI specialists included in the interviews were aware of primary care pathways; however, it appeared that GI specialists believed that more awareness is needed among PCPs.

*I think creating more awareness would be helpful. I don't know, like how they're reaching out to different PCNs about these, but there are definitely people like GPs and some good GPs out there who aren't aware that these pathways exist and that they're really good resources. [GI specialist, ARP]*

*(2) Benefits of CAT*. GI specialists noticed a reduction in their waitlist because of implementing primary care support tools.

*Like I'm a big fan and proponent of, you know, central triage. I think it creates more equity and just much more standardization. And I think having the pathways and having central triage, I think those are good ways to kind of streamline and consults cut down on unnecessary wait times, and just overall improve the efficiency within the system. [GI specialist, ARP]*

GI specialists perceived that access has been improved as a result of implementing CAT.

*I have seen that these patients sometimes have been jerked around as different doctors have passed them. By using central triage, it makes a patient have to be seen by a GI specialist eventually. So I've seen that as actually beneficial. [GI specialist, Fee for Service]*

GI specialists reported that CAT not only provided fair access to GI specialists for patients but also ensured steady referrals for GI specialists.

*You're part of a system where there's like a flood of referrals, right? So I know in COVID-19 and Calgary, some of the doctors who are getting referrals from family doctors, had no referrals, right? They had no work, right? Whereas if they're part of the central triage program, you get work no matter what happens, right? So that would ensure that you have a steady referral base to get your patient seen. [GI specialist, Fee for Service]*

*(3) Challenges with CAT*. There are perceived biases within CAT:

*The issue with it is a bit of the autonomy goes away. So, some of these referrals I have no interest in seeing, right, somebody who's had abdominal pain for 30 years. But the central triage system will basically force you to have to take those referrals. [GI specialist, Fee for Service]*

*I think there has always been some concern from the fee-for-service physicians that the academic people are going to kind of cherry-pick off interesting cases or rare cases or organic cases. And then the community fee for service physicians will be left dealing with nothing but IBS and things like that. [GI specialist, ARP]*

*(4) Checklist to Standardize CAT Process*. Participants mentioned CAT processes were subjective:

*There's no standard checklist that's been shared with me as yet. There are some criteria for the prioritization of urgency levels. [GI specialist, Fee for Service]*

*(5) Clinical Audits to Promote Best Practices*. Participants suggested an audit of endoscopic procedures may help to examine and then reduce unnecessary procedures:

*I think in gastroscopy, 80% of the time we don't find anything anyways, right? So that would be the key if you audit them all and you find out that a lot of people are doing procedures that aren't necessary then that would be a reason to get rid of the waiting list. [GI specialist, Fee for Service]*

Similarly, an audit of PCP referrals may help reduce unnecessary referrals. This inquiry could be used for education with PCPs. Understanding referrals may shed light on drivers of higher referral rates.

*And if you find that Dr. X is the person who's always referring that well, then maybe actually supporting them. It might be they have a really complex patient population that they serve. Like the low socioeconomic status or English language resources, or they're from a particular community where actually the pathway just isn't appropriate for them. [GI specialist, Fee for Service]*

## 4. Discussion

Overall, this mixed method study demonstrates some important insights into mechanisms built in Alberta to improve access to GI care and highlights important strengths associated with the implementation of CAT and primary care pathways from both PCPs and GI specialists. Some of the most important contributions from CAT include an effective means of standardizing care, with assurance of transparency and equity in accessing specialty care. For primary care pathways, marked advantages include the empowerment of PCPs to manage patients in their PCM using best-evidence method, practical algorithms with increased appropriateness, and timeliness of investigations and patient management. Despite significant existing complexities within the health system, CAT and primary care supports may facilitate improved system integration, standardize communication, and enhance collaboration, aimed to improve health outcomes for patients. The inclusion of perceptions from both PCPs and GI specialists about CAT and primary care supports such as care pathways, telephone advice, and CAT provide substantive and balanced insight into the variable supports built to improve system integration.

PCPs reported their use of pathways was mainly determined by the increased likelihood of having their referral accepted by the specialist with CAT (39.4% strong agreement and 42.4% agreement). Nevertheless, PCPs also recognized pathways offer evidence-based guidance to evaluate patients with digestive complaints and reduced the number of patients for whom the referral was necessary. This is consistent with the extant published research study, demonstrating reduced wait times and improved patient outcomes with pathway implementation (or similar clinical algorithms). Srivastava et al. [20] reported the use of a nonalcoholic fatty liver disease pathway increased the detection of advanced fibrosis and cirrhosis (OR 4.23, 95% CI: 1.52-12.25, *p*=0.006), with a decrease in unnecessary referrals (OR 0.23; 95% CI: 0.66–0.82, *p*=0.006). PCPs expressed the usefulness of the pathways in conjunction with CAT as a means of ensuring fairness and equitable access to tertiary care and endoscopy. PCPs also reported difficulty in managing patient expectations of a referral. There are limitations; however, several PCPs expressed caution with the use of pathways for patients accessing walk-in clinics given the lack of certain return to care or follow-up. Similarly, for vulnerable patients, such as those experiencing homelessness or addiction, who cannot easily undergo the investigation and follow-up to complete a pathway limited safe use in this context. Thus, a strong theme emerged from the PCP surveys and interviews that pathways could not effectively function in a “one-size-fits-all” capacity, and a more nuanced approach to ensure appropriate follow through, facilitated by ongoing communication and collaboration to optimize use. In addition, GI specialist were hopeful standardized approaches to triage and would be implemented with CAT, consistent with a previously published scoping review on referral criteria for gastroenterology, recommending the “development of a prioritization referral tool…” as well as “for primary care providers, (a) tool (which) would help to standardize the referral process, reducing the frustration of multiple forms and referral requirements” [21].

GI specialists included in this study reported satisfaction with both the primary care pathways and the implementation of CAT in reducing unnecessary referrals. In the survey and qualitative interviews, GI specialists believed pathways would be beneficial in strengthening the medical home model, facilitate best-evidence management to expediate care and thus reduce wait time and resource expenditure for patients referred to their clinics for specialty care. However, variability among specialists was observed among different remuneration models: FFS model physicians were less likely to participate in CAT than those in an ARP model. There was little agreement with the statements “CAT is an efficient use of health system resources” and “CAT improves access for patients” between FFS and ARP GI specialists. The remuneration model itself is unlikely the only explanation for variable perceptions about CAT and primary care tools between these groups. FFS specialists, for example, are independent contractors, guided largely by the principle or value of autonomy. Skepticism regarding the benefits of CAT is therefore not uncommon, and perceived loss of control over the referral triage process may contribute to reservations around fairness or consistency. Alternatively, the ARP model specialist's professional responsibilities are less tied to referral volume [22]. Both groups, however, identified measurement of referral demand and wait times as paramount.

This study had three main limitations. Firstly, the response rate to the survey was low and we had a small survey sample from both PCPs and GI specialists. Although the PCP survey sample was small, a number of in-depth qualitative interviews provided rich data regarding their views on primary care tools' implementation. The GI specialist sample was small in both surveys and interviews, so findings should be interpreted with caution; however, their insights were highly valuable to understand and improve triage processes and understand limitations. Secondly, the physician participation (for both PCPs and GI specialists) hailed mainly from urban areas, thus may have a more “urban-centric” view of pathway implementation and impacts. Rural patients face significant challenges accessing healthcare and may face worse health outcomes. Training rural full-scope family physicians in endoscopic procedures may help address these needs [9, 23]. Family physicians (PCPs) in our study also revealed the potential benefits of training primary care providers in endoscopic procedures, yet noted access to use endoscopy suites remain limited.

Future studies are required to focus on further optimizing referral appropriateness, standardizing CAT services across urban and rural sites, and increasing capacity to improve patient access to GI-related health services.

## 5. Conclusions and Recommendations

Primary care pathways are valued and widely used by PCPs in Alberta; however, their implementation continues to face a number of challenges. Further support including education and training for PCPs in pathway use may ease these barriers. CAT services play an important role to ensure fair and equitable access to GI specialists; however, the system is not perfect and challenges exist for both PCPs and GI specialists. Improved communication and collaboration between primary and specialty care is core to better system integration.

Significant variation exists between FSS and ARP GI specialists regarding their perceptions of the benefits of CAT and primary care supports. ARP specialists have more positive views regarding the benefits of CAT and primary care supports. Overall, improvement in CAT processes and increased awareness of primary care supports may significantly reduce the number of low yield referrals and endoscopies.

Summary recommendations for successful implementation of primary care support tools for improving access to specialists include the following:

- Clear, evidence-based triage processes should be transparently implemented to ensure the sickest patients will be consistently prioritized
- Data collection and measurement to quantify, characterize, and inform providers regarding low yield and avoidable referrals are important to improve the referral process for primary care
- Engagement and collaboration among all GI specialists including FFS and ARPs among others in leading and guiding CAT are important for successful and sustainability of CAT and primary care support tools

## Figures and Tables

**Table 1:** Demographics of PCPs who participated in the survey (*N* = 36).

Variables	Frequencies (%) (*n*)
Area of practice	
North	0.0 (0)
Edmonton	25.0 (9)
Central	5.6 (2)
Calgary	61.1 (22)
South	8.3 (3)
PCN member	
Yes	86.1 (31)
No	13.9 (5)
Years in practice	
0–5	14.7 (5)
6–10	38.2 (13)
11–15	11.8 (4)
16–20	5.9 (2)
21+	29.4 (10)
Full-time or part-time practice	
Full-time	73.5 (25)
Part-time	26.5 (9)
Gender	
Male	26.5 (9)
Female	73.5 (25)
Prefer not to say	0.0 (0)
My gender is not listed here	0.0 (0)

**Table 2:** Demographics of GI specialists who participated in the survey (*N* = 28).

Variables	Frequencies % (*n*)
Years in practice	
0–5	21.4 (6)
6–10	28.6 (8)
11–15	21.4 (6)
16–20	7.1 (2)
21+	21.4 (6)
Full-time vs. part-time practice	
Full time	96.4 (27)
Part time	3.6 (1)
Remuneration model	
Alternative relationship plan	53.6 (15)
Fee for service	42.9 (12)
Prefer not to say	3.6 (1)
Gender	
Male	50.0 (14)
Female	39.2 (11)
Prefer not to say	7.1 (2)
My gender is not listed here	3.5 (1)
Participation in a CAT program	
Yes	73.9 (17)
No, but I previously participated in a central triage program	8.8 (2)
No, I have never participated in a central triage program	17.4 (4)

**Table 3:** Comparing perceptions about the benefits of CAT between GI specialists working on FFS model and ARP model.

Do you agree that the following elements are benefits of CAT?	FFS-GI specialists mean (SD)	ARP-GI specialists mean (SD)	*T*-test statistics	*p* value
Specialists all use the same criteria for accepting referrals	3.18 (1.5) *n* = 11	3.21 (1.5) *n* = 14	0.06	0.95
CAT improves the quality of referrals	3.27 (1.0) *n* = 11	3.57 (1.2) *n* = 14	0.65	0.52
CAT is an efficient use of health system resources	3.36 (1.1) *n* = 11	4.21 (0.7) *n* = 14	2.20	0.04^*∗*^
CAT reduces physician workload	3.18 (1.1) *n* = 11	3.50 (1.1) *n* = 14	0.73	0.47
PCPs prefer to use central triage	2.55 (1.0) *n* = 11	3.31 (1.1) *n* = 13	1.66	0.11
CAT ensures that patients with the greatest need are prioritized	3.45 (0.9) *n* = 11	3.93 (0.9) *n* = 14	1.22	0.24
CAT optimizes use of endoscopy resources	3.00 (0.9) *n* = 11	3.64 (0.9) *n* = 14	1.75	0.09
CAT improves access for patients	2.64 (1.0) *n* = 11	3.50 (1.0) *n* = 14	2.10	0.04^*∗*^
CAT provides a standardized approach to measure and report on referrals and wait times	3.91 (0.9) *n* = 11	3.86 (0.8) *n* = 14	0.14	0.89

Score: 1 = strongly disagree, 2 = disagree, 3 = neither agree nor disagree, 4 = agree, and 5 = strongly agree. ^*∗*^Statistically significant difference. Pearson chi-square value.

**Table 4:** Comparing GI specialists working on FFS model versus ARP model regarding their perceptions about pathway development and utilization in Alberta.

Do you agree that the following principles are important for effective pathway development and utilization in Alberta?	Fee for service-GI specialists mean (SD)	ARP-GI specialists mean (SD)	*T*-test statistics	*p* value
Pathways are codeveloped between primary and specialty care providers	4.36 (0.5) *n* = 11	4.85 (0.3) *n* = 13	2.68	0.01^*∗*^
Pathways are evidence-informed and updated on a regular basis	4.40 (0.5) *n* = 10	4.77 (0.4) *n* = 13	1.85	0.07
All specialties use a common format for pathways	4.09 (0.5) *n* = 11	4.67 (0.5) *n* = 12	2.67	0.01^*∗*^
Pathways across all specialties are available in one location	4.22 (0.3) *n* = 9	4.83 (0.3) *n* = 12	3.36	<0.01^*∗*^
Where a pathway is available, use of PCPs required before referral will be accepted by specialists	3.82 (0.8) *n* = 11	4.42 (0.9) *n* = 12	1.65	0.12
Pathways include relevant patient education/self-management resources	4.27 (0.6) *n* = 11	4.67 (0.5) *n* = 12	1.63	0.11
Multidisciplinary support is available in the PCM	4.09 (0.8) *n* = 11	4.58 (0.6) *n* = 12	1.57	0.13
Specialist advice is available to support PCPs in using pathways	4.00 (0.6) *n* = 11	4.75 (0.4) *n* = 12	3.29	<0.01^*∗*^
Providers have a mechanism to provide feedback on pathways	4.09 (0.5) *n* = 11	4.75 (0.4) *n* = 12	3.16	<0.01^*∗*^
Regular evaluation and reporting of outcomes	4.36 (0.5) *n* = 11	4.83 (0.3) *n* = 12	2.51	0.02^*∗*^

Score: 1 = strongly disagree, 2 = disagree, 3 = neither agree nor disagree, 4 = agree, and 5 = strongly agree. ^*∗*^Statistically significant difference.

## Data Availability

The data used to support the findings of the study are available from the corresponding author upon request.

## Abbreviations

AH: Alberta Health
AHS: Alberta Health Services
ARP: Alternative relationship plan
CAT: Centralized access and triage
DHSCN: Digestive health strategic clinical network
IBS: Irritable bowel syndrome
PCP: Primary care provider
GI: Gastrointestinal
CAG: Canadian Association of Gastroenterology
PCM: Patient-centered medical home
PCN: Primary care network
ED: Emergency department
GERD: Gastroesophageal reflux disease
NAFLD: Nonalcoholic fatty liver disease
FFS: Fee for service.
